# Heme Oxygenase-1 Has a Greater Effect on Melanoma Stem Cell Properties Than the Expression of Melanoma-Initiating Cell Markers

**DOI:** 10.3390/ijms23073596

**Published:** 2022-03-25

**Authors:** Anna Kusienicka, Karolina Bukowska-Strakova, Maciej Cieśla, Witold Norbert Nowak, Iwona Bronisz-Budzyńska, Agnieszka Seretny, Monika Żukowska, Mateusz Jeż, Rościsław Krutyhołowa, Hevidar Taha, Neli Kachamakova-Trojanowska, Halina Waś, Claudine Kieda, Alicja Józkowicz

**Affiliations:** 1 Department of Medical Biotechnology, Faculty of Biophysics, Biochemistry and Biotechnology, Jagiellonian University, 30-387 Krakow, Poland; k.bukowska-strakova@uj.edu.pl (K.B.-S.); m.ciesla@imol.institute (M.C.); witold.nowak@uj.edu.pl (W.N.N.); iwona.bronisz-budzynska@doctoral.uj.edu.pl (I.B.-B.); a.seretny@dkfz.de (A.S.); monika.zukowska@alumni.uj.edu.pl (M.Ż.); mateusz.jez@med.lu.se (M.J.); rostyslav.krutyholova@doctoral.uj.edu.pl (R.K.); hevidar.taha@uod.ac (H.T.); neli.kachamakova-trojanowska@uj.edu.pl (N.K.-T.); hwas@wim.mil.pl (H.W.); 2 Department of Clinical Immunology and Transplantology, Institute of Pediatrics, Jagiellonian University Medical College, 30-663 Krakow, Poland; 3 Basic Sciences Department, College of Agricultural Engineering Sciences, University of Duhok, Zakho Street 38, Duhok 1006 AJ, Kurdistan Region, Iraq; 4 Laboratory of Molecular Oncology and Innovative Therapies, Military Institute of Medicine, 04-141 Warsaw, Poland; ckieda@wim.mil.pl; 5 Centre for Molecular Biophysics, UPR4301 CNRS, 45071 Orleans, France

**Keywords:** heme oxygenase-1, melanoma-initiating cells, cancer stem cells, cancer cell heterogeneity

## Abstract

Melanoma-initiating cells (MICs) contribute to the tumorigenicity and heterogeneity of melanoma. MICs are identified by surface and functional markers and have been shown to display cancer stem cell (CSC) properties. However, the existence of MICs that follow the hierarchical CSC model has been questioned by studies showing that single unselected melanoma cells are highly tumorigenic in xenotransplantation assays. Herein, we characterize cells expressing MIC markers (CD20, CD24, CD133, Sca-1, ABCB1, ABCB5, ALDH^high^) in the B16-F10 murine melanoma cell line. We use flow cytometric phenotyping, single-cell sorting followed by in vitro clonogenic assays, and syngeneic in vivo serial transplantation assays to demonstrate that the expression of MIC markers does not select CSC-like cells in this cell line. Previously, our group showed that heme-degrading enzyme heme oxygenase-1 (HO-1) can be upregulated in melanoma and increase its aggressiveness. Here, we show that HO-1 activity is important for non-adherent growth of melanoma and HO-1 overexpression enhances the vasculogenic mimicry potential, which can be considered protumorigenic activity. However, HO-1 overexpression decreases clone formation in vitro and serial tumor initiation in vivo. Thus, HO-1 plays a dual role in melanoma, improving the progression of growing tumors but reducing the risk of melanoma initiation.

## 1. Introduction

Melanoma is a highly aggressive skin cancer of melanocytic origin. In late-stage disease, the overall 5-year survival is very low, around 30% [1], and the prognosis is even worse when melanoma affects mucosal surfaces [2,3]. Despite the introduction of new treatments (e.g., targeted BRAF^V600E/V600K^ therapies or immune checkpoint inhibitors) [4], late-stage melanoma is practically incurable and displays high therapy resistance. This therapy resistance occurs largely due to the exceptionally high heterogeneity of melanoma [5], which is attributed to the presence of the cancer stem cells (CSCs), among others. CSCs can self-renew through asymmetrical or symmetrical divisions and give rise to all lineages of more differentiated, proliferating tumor cells that maintain tumor growth [6]. CSCs are thought to arise from mutations in normal stem cells, transiently amplifying progenitor cells [7], or from non-CSCs through de-differentiation [8]. The most recent model assumes that CSCs are a dynamic population that transiently display CSC-like phenotype depending on the signals from the environment [9].

In melanoma, cells with CSC properties are called melanoma-initiating cells (MICs) and were characterized by the expression of surface markers including CD20 [10], ABCB5 [11,12,13], CD133 [14,15], and CD271 [16,17,18,19] and functional features such as the high activity of aldehyde dehydrogenases (ALDHs) [20], ability to efflux Hoechst 33342 (so-called “side population”) [21], and slow proliferation rate [22,23,24]. Although many MIC subsets have been identified, the existence and tumorigenic potential of MICs have been controversial. In 2008, only three years after the identification of the first MIC subpopulation, Quintana et al. published a paper in which they undermined the existence of CSCs in human melanoma [25]. They proved that changes in xenotransplantation assay can increase the observed tumorigenicity of melanoma [25]. As a continuation of this study, the same group performed a broad analysis of the tumorigenic capacity of melanoma cells expressing different MIC markers and found that melanoma cells are highly tumorigenic regardless of the MIC marker expression when transplanted to the NSG mice [26]. They showed that melanoma is not hierarchically organized and melanoma cells can switch the expression of MIC markers on and off, indicating that heterogeneity of melanoma might be caused by phenotypic plasticity of the cells [26]. Other reports also highlighted that previously described MIC markers (such as ALDH, CD133, and CD271) do not necessarily mark the cells with increased tumorigenic potential [27,28,29]. All these studies emphasized the role of the immune system and CSC niche to be crucial for the regulation of CSC behavior. Thus, CSC experiments that are devoid of the niche context may not detect all physiological functions of these cells [30]. Having this in mind, there is a need for further characterization of the biology of MICs in syngeneic systems. Moreover, as cells with CSC properties are essential for melanoma heterogeneity and chemoresistance, it is crucial to define the factors that regulate their behavior.

One such factor is the activity of heme oxygenase 1 (HO-1, encoded by *Hmox-1* gene), an inducible form of heme-degrading enzyme that plays an important role in many physiological and pathophysiological settings, including cell response to oxidative stress, cell differentiation, and tumorigenesis. It catalyzes the degradation of heme to carbon monoxide (CO), ferrous ions (Fe^2+^), and biliverdin [31]. Activated HO-1 reduces oxidative stress, modulates immune response, decreases apoptosis, and affects angiogenesis [31]. However, these cytoprotective functions of HO-1 can become problematic when hijacked by tumor cells. HO-1 is often overexpressed in cancerous cells when compared to the corresponding healthy tissues [32,33] and is further induced by chemo-, radio-, and photodynamic therapies [34]. Its expression can be also induced by some oncogenes, including BCR/ABL [35] and Pax3/7-FoxO1 [36]. Interestingly, the outcome of HO-1 activity in tumors is cell-type-dependent [37]. The existing data on the role of HO-1 in melanoma show that this enzyme acts in favor of this cancer. The presence of a shorter HO-1 promoter associated with increased HO-1 expression is more frequent in melanoma patients than in healthy individuals, which highlights the potential role of high expression of HO-1 in this cancer [38]. Moreover, our data obtained on B16-F10 murine melanoma showed that overexpression of HO-1 increases proliferation rate, improves protection against oxidative stress, and enhances the aggressiveness of melanoma cells in vivo [39]. On top of that, multiple reports show the significance of HO-1 activity in melanoma resistance against therapies [40,41,42,43]. HO-1 drives the resistance of BRAF^V600E^ mutated melanoma cells against vemurafenib treatment [44] and can directly interact with BRAF protein, leading to the activation of CDK2/cyclin E-dependent induction of cell proliferation [45]. Altogether, available literature highlights the importance of HO-1 in melanoma aggressiveness and resistance to treatments. However, nothing is known about the possible effects of HO-1 in MICs.

The goal of the current study is to characterize the effect of HO-1 overexpression on the activities of MICs. Based on our previous study where we have shown that HO-1 increases the aggressiveness of melanoma in growing tumors, we hypothesized that HO-1 might also affect the biology of MICs. Using the B16-F10 cell line overexpressing HO-1, we studied the effect of this enzyme on MIC properties, including non-adherent growth, vasculogenic mimicry, clonogenicity, and tumorigenicity. In vivo studies were performed using the syngeneic C57BL/6 mouse strain which assured the intact immune system, crucial for the accurate assessment of MIC tumorigenicity. As a result, we identified several MIC subsets in the B16-F10 cell line and demonstrated that HO-1 overexpression, and not the MIC markers expression, predominantly affected melanoma clonogenicity in vitro and tumorigenicity in vivo in a syngeneic transplantation assay.

## 2. Results

### 2.1. HO-1 Affects Non-Adherent Growth, Vasculogenic Mimicry, and Expression of CSC-Associated Genes in B16-F10 Melanoma Cells

**Figure 1:**
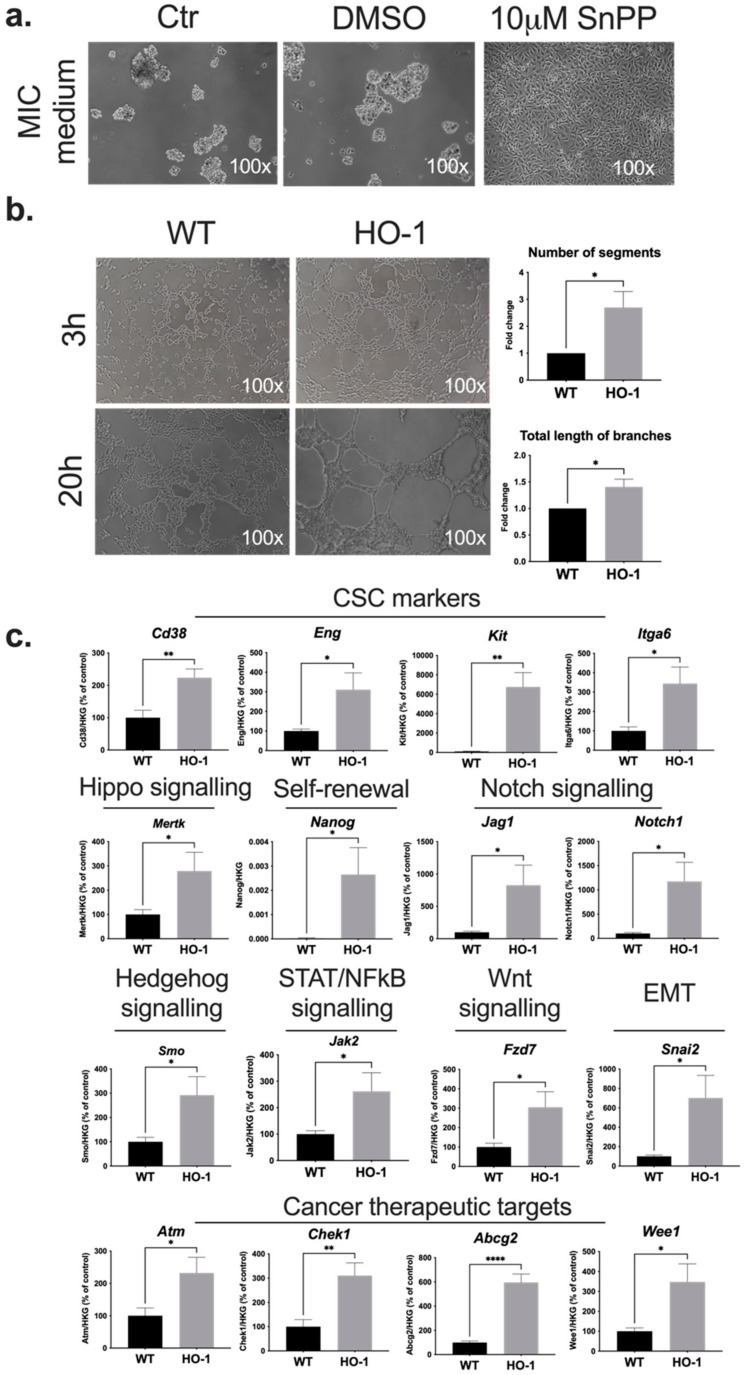
HO-1 overexpression affects CSC-like properties of B16-F10 melanoma cells. (**a**) Representative microscopic pictures of B16-F10 cells cultured 7 days in MIC medium: Ctr, MIC medium; DMSO, MIC medium supplemented with DMSO as a diluent control; 10 µM SnPP, MIC medium supplemented with 10 µM tin protoporphyrin IX (n = 4). (**b**) In vitro Matrigel-based tube formation assay. B16-F10 WT and HO-1 cells were seeded on Matrigel matrix and monitored 3 h and 20 h after seeding. Image J analysis using Angiogenesis Analyzer plugin was performed using pictures taken 3 h after cell seeding (n = 5, each bar represents mean fold change + SEM). (**c**) RT^2^Profiler Array (Qiagen) of CSC-associated genes performed on B16-F10 WT and HO-1 cells (HKGs, housekeeping genes; n = 4–5; each bar represents mean + SEM; * *p* < 0.05, ** *p* < 0.01, **** *p* < 0.0001).

HO-1 is often overexpressed in cancer cells and induced by cancer therapies [28,29,30]. In this study, we used the B16-F10 murine melanoma cells engineered to stably overexpress HO-1 together with Luc transgene (Figure S1) or with GFP-Luc transgene [46]. Engineered control cells expressed reporter proteins only. The HO-1 overexpression was confirmed at mRNA, protein, and enzymatic activity levels (Figure S1). As in vitro research on CSC properties requires the usage of chemically defined serum-free media [47], we cultured B16-F10 cells in melanoma-initiating cell (MIC) medium [48]. After 7 days melanoma cells started to grow as non-adherent melanospheres (Figure 1a). Interestingly, when heme oxygenase inhibitor, tin protoporphyrin IX (SnPP), was added, we observed inhibition of non-adherent growth, indicating an important role of heme oxygenase activity in this CSC feature (Figure 1a).

HO-1 is a known proangiogenic factor that promotes vascularization in melanoma tumors [39]. Therefore, using in vitro tube formation assay, we sought to identify the effects of HO-1 on vasculogenic mimicry (VM), as MICs are thought to contribute to this process of formation of vessel-like structures [49]. To assess the VM potential of melanoma cells, we seeded the control B16-F10 cells (wild type (WT)) and HO-1-overexpressing cells on a Matrigel matrix [50]. After 3 h, the cells had already started to form pseudo-tubule structures that were even more pronounced in the later time point (20 h), especially for HO-1 cells (Figure 1b). The quantitative analysis using Angiogenesis Analyzer (ImageJ software) revealed that the formation of tubes was significantly more pronounced in HO-1-overexpressing melanoma cells (Figure 1b).

Seeing that in melanoma cells HO-1 influenced some of the functional properties typical for CSCs, we checked if overexpression of HO-1 modifies the expression of genes associated with CSC function. We used the RT^2^Profiler Array (Qiagen), the system that enables the analysis of many pre-determined CSC-associated genes, including 84 genes of interest and 5 housekeeping genes (HKGs). We found that HO-1 overexpression led to the upregulation of several CSC markers (*Cd38*, *Eng*, *Kit*, *Itga6*); genes involved in essential signaling pathways (Hippo, Notch, Hedgehog, STAT/NFκB, Wnt), self-renewal (*Nanog*), and epithelial-to-mesenchymal transition (*Snai2*); and cancer therapeutic target genes (*Atm*, *Chek1*, *Abcg2*, *Wee1*) (Figure 1c). This result further highlighted the importance of HO-1 in the CSC-associated properties of melanoma cells.

### 2.2. B16-F10 Cell Line Contains Cell Fractions That Express Cell Surface and Functional MIC Markers

**Figure 2:**
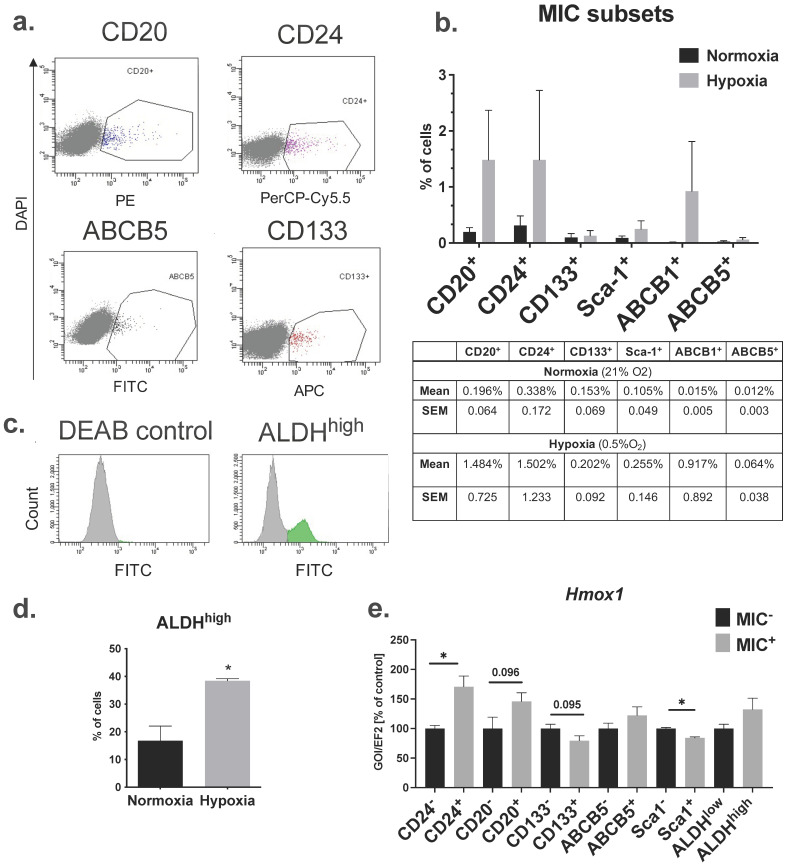
Frequency of cells expressing MIC markers in B16-F10 murine melanoma cell line. (**a**) Representative flow cytometry analysis (dot plots) of surface MIC markers. (**b**) Flow cytometry analysis of MIC surface markers of B16-F10 cells cultured 48 h in normoxia (21% O_2_) or hypoxia (0.5% O_2_) (n = 2–3, each bar represents mean + SEM). (**c**) Representative histograms of ALDH^high^ cells detected with ALDEFLUOR kit. (**d**) Flow cytometry analysis of ALDH^high^ cells cultured in normoxia or hypoxia (n = 3, each bar represents mean + SEM; * *p* < 0.05). (**e**) Expression of *Hmox-1* mRNA levels in MIC subsets. qRT-PCR on 50 sorted cells after pre-amplification with the AmpliGrid system. GOI, gene of interest; *Ef2* was used as a reference gene (each bar represents mean from 3–6 sorted samples + SEM; * *p* < 0.05).

The non-adherent growth of B16-F10 melanoma cells in MIC medium and ability to form VM-like structures on Matrigel suggested that this cell line could contain cells of CSC phenotype. Therefore, in the next step, we checked if the B16-F10 murine melanoma cell line contains subpopulations with MIC signatures. As melanoma cell heterogeneity can be driven by hypoxia [51], we cultured the cells for 48 h under normoxic (21% O_2_) or hypoxic (0.5% O_2_) conditions. Flow cytometry analysis showed that small fractions of B16-F10 cells express the MIC surface markers CD20, CD24, CD133, Sca-1, ABCB1, and ABCB5 (Figure 2a,b). Percentages of cells expressing the single MIC surface markers (MIC^+^ cells) in normoxia were very low, not exceeding 0.5% even for the most frequent, namely CD24^+^ and CD20^+^, fractions (Figure 2b). In hypoxia, the frequency of MIC^+^ cells was slightly increased but still low (Figure 2b). We did not observe any distinguishable subpopulations that co-expressed investigated MIC markers (data not shown), which suggests that MIC^+^ are a heterogeneous population of cells.

Additionally, we used a functional marker, high aldehyde dehydrogenase activity (ALDH^high^), to distinguish a potential subpopulation of MICs (Figure 2c). ALDH^high^ cells were much more frequent than cells expressing MIC cell surface markers, and their frequency increased significantly in hypoxia (Figure 2d). However, it should be noted that percentages of cells with specific surface markers and ALDH^high^ cells can vary between experiments, which we observed within the course of this study.

Low expression of melanoma-associated antigens (MAAs) was shown to mark the de-differentiated state of MICs [16,52]. We compared the expression of known MAAs, namely tyrosinase (*Tyr*), glycoprotein 100 (*Gp100* also known as *Pmel*), melanoma antigen recognized by T cells (*Mart-1*), and microphthalmia-associated transcription factor (*Mitf*), in the MIC^−^ and MIC^+^ cells. Data revealed that expression of MAAs did not differ significantly between MIC^+^ and MIC^−^ cells and there was no clear expression pattern of MAAs in different MIC^+^ subpopulations (Figure S2).

As the development of melanoma is facilitated by a high expression level of HO-1 [38,39], we compared the expression of *Hmox-1* in MIC^+^ subsets and MIC^−^ cells. The set of qRT-PCRs showed that *Hmox-1* is differently regulated in different MIC^+^ subsets (Figure 2e). Namely, *Hmox-1* level was significantly increased in CD24^+^ cells and showed a tendency to be increased in CD20^+^ cells, but at the same time, its level was slightly but significantly decreased in Sca-1^+^ cells and showed a tendency to be decreased in CD133^+^ cells (Figure 2e). No differences were found in ABCB5^+^ and ALDH^high^ (Figure 2e). This suggests that HO-1 up- or downregulation on mRNA level is not a determinant of the expression of MIC markers.

### 2.3. Overexpression of HO-1 Decreases the Clonogenic Potential of Melanoma Cells In Vitro

**Figure 3:**
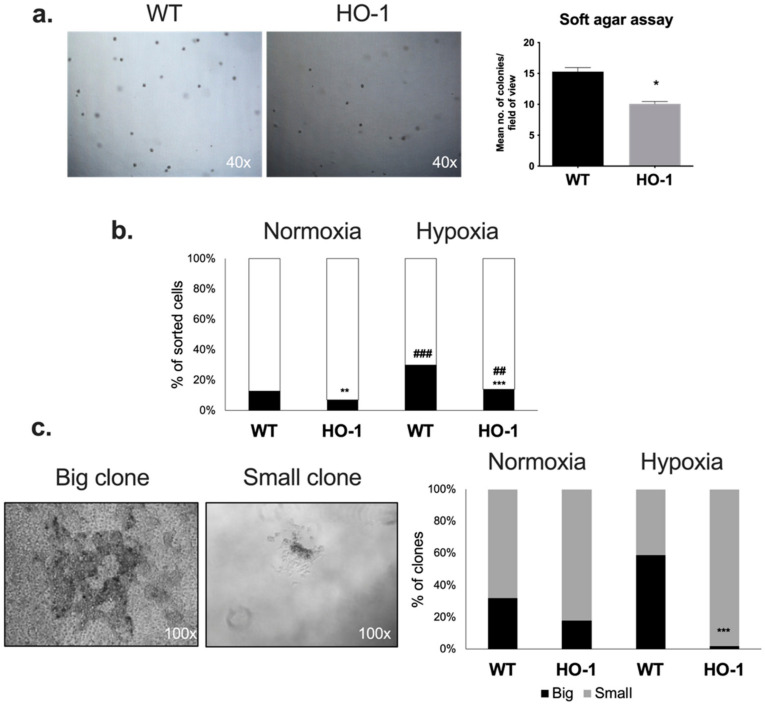
Effect of HO-1 overexpression on the clonogenic potential of B16-F10 melanoma cells. (**a**) Soft agar assay performed using WT and HO-1-overexpressing cells. Microscopic view of melanoma spheres 7 days after seeding. Numbers of spheres were calculated by two independent scientists (n = 2, each bar represents mean + SEM; * *p* < 0.05). (**b**) Percentage of cells that formed stable clones 13 days after single-cell sorting (n = 239–624, Fisher exact test; ** *p* < 0.01, *** *p* < 0.001 versus WT cells; ## *p* < 0.01, ### *p* < 0.001 versus normoxia). (**c**) Size of clones formed by WT and HO-1 melanoma cells in normoxia and hypoxia, 13 days after single-cell sorting (n = 39–76, Fisher exact test; *** *p* < 0.001 versus WT cells).

Because the enzymatic activity of heme oxygenase was required for non-adherent growth (Figure 1a), in the next step we checked whether HO-1 overexpression can increase the clonogenic potential of melanoma cells, considered as a feature of CSCs [53]. For this purpose, we used a soft agar assay that allows the study of non-adherent clonogenic growth. Unexpectedly, we found out that HO-1 overexpression decreases the ability of B16-F10 cells to form spheres in the soft agar (Figure 3a).

To verify this observation, we additionally analyzed the formation of clones by single-cell sorted WT and HO-1 B16-F10 melanoma cells cultured in MIC medium in normoxia (21% O_2_) or hypoxia (0.5% O_2_). In this experimental setting, only a minority of B16-F10 melanoma cells (13%) were able to form stable clones in normoxia, but this fraction was higher in hypoxia (30%) (Figure 3b).

In accordance with the results of the soft agar assay, overexpression of HO-1 decreased the ability of single cells to form clones, both in normoxia (7%) and hypoxia (14%) (Figure 3b). Microscopic observations revealed that B16-F10 clones differed in some morphological features including size (Figure 3c). Most of the clones formed by B16-F10 cells cultured in normoxia were classified as small, regardless of HO-1 status. The significant differences were visible in hypoxia: 13 days after sorting, approximately 60% of clones formed by WT cells were big, whereas HO-1-overexpressing cells formed almost exclusively small clones, which might reflect their lower proliferation rate in this experimental setting (Figure 3c).

### 2.4. CD20 Expression and High ALDH Activity Do Not Affect the Clonogenic Potential of Murine Melanoma Cells

**Figure 4:**
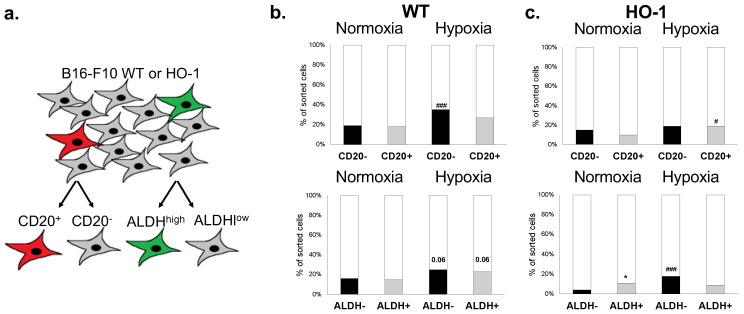
Formation of clones by single-cell sorted MIC^+^ melanoma cells cultured in normoxia or hypoxia. (**a**) Scheme of sorting; comparison of MIC clone formation in (**b**) WT and (**c**) HO-1 cells 13 days after sorting (n = 116–216, Fisher exact test; * *p* < 0.05 versus WT-Luc cells, # *p* < 0.05, ### *p* < 0.001 versus normoxia).

The same clonogenic test was performed for MIC fractions. For this purpose, we chose two MIC subsets: cells expressing surface antigen CD20 and cells displaying a functional marker—high ALDH activity (Figure 4a). The result showed tendencies similar to those observed for bulk cells, towards the increased clonogenicity in hypoxia and reduced clone formation by HO-1-overexpressing cells (Figure 4b,c). Importantly, neither expression of CD20 nor ALDH activity influenced the clonogenic potential of WT (Figure 4b) and HO-1 (Figure 4c) melanoma cell lines. Only HO-1 cells with high ALDH activity increased clone formation in normoxia when compared to HO-1 ALDH^low^ cells (Figure 4c), but this might be an indirect effect of a lower than normally observed clonogenicity of control cells. These results show that phenotypic or functional MIC markers in murine melanoma cell line do not identify cell fractions with increased clonogenic potential. Consistent with what was observed for bulk populations, HO-1 overexpression led to the formation of smaller clones in hypoxia, regardless of the MIC expression status (Figure S3).

### 2.5. Progeny of MIC^+^ Cells Reconstitute the Heterogeneity of the Parental Cell Line

**Figure 5:**
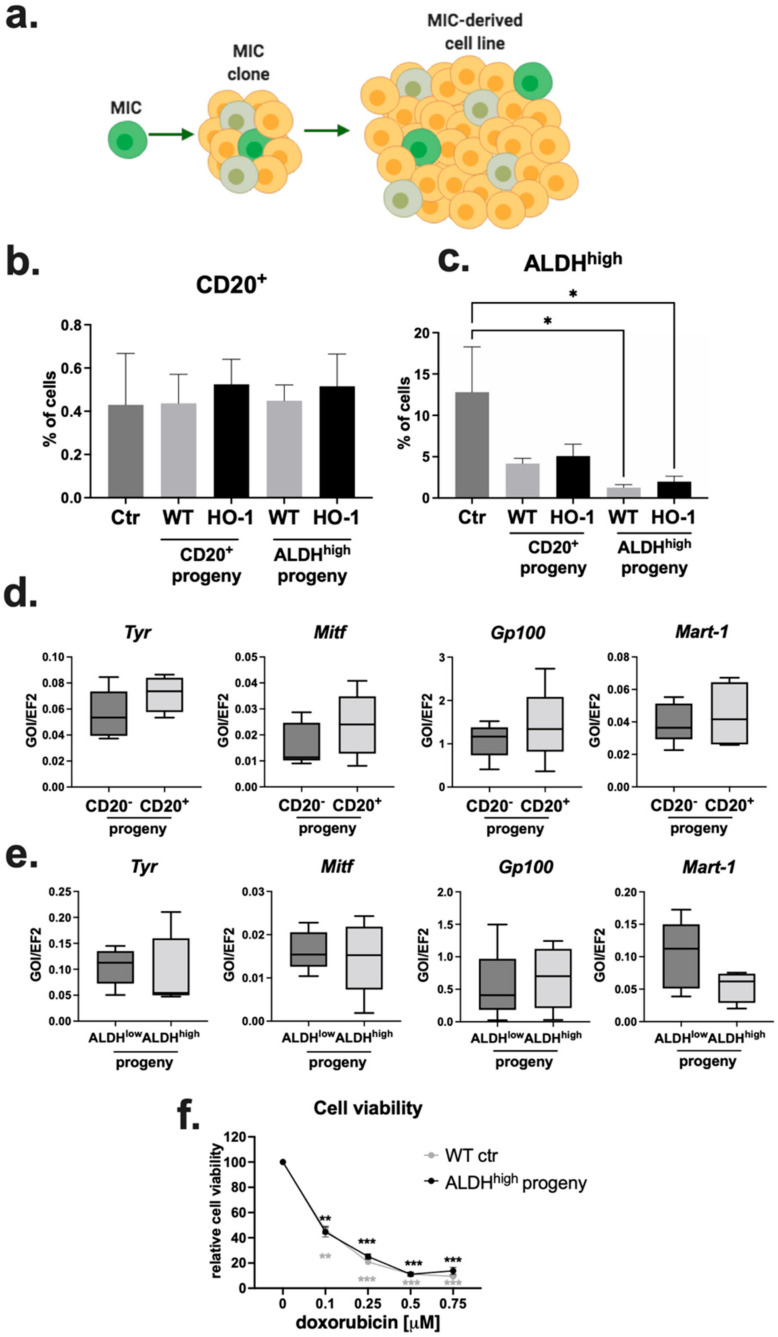
Characterization of MIC-derived clonogenic cell lines. (**a**) The approach used to obtain single-cell derived cell lines, illustration created using BioRender. Flow cytometry analysis of the frequency of (**b**) CD20^+^ and (**c**) ALDH^high^ cells in the parental B16-F10 WT cell line (Ctr) and CD20^+^-derived and ALDH^high^-derived clonogenic cell lines (n = 3–5, each bar represents mean + SEM, * *p* < 0.05). qRT-PCR analysis of the expression of MAAs in cell lines derived from single (**d**) CD20^−^/CD20^+^ cells and (**e**) ALDH^low^/ALDH^high^ cells. *Ef2* was used as a housekeeping control (n = 5, box and whisker plots). (**f**) MTT reduction assay performed on ALDH^high^ progeny and control cells treated with doxorubicin for 24 h (n = 3, each point represents mean ± SEM; ** *p* < 0.01, *** *p* < 0.001 versus untreated cells).

Clones formed by MIC^+^ cells continued to grow, giving rise to progeny-derived cell lines (Figure 5a). Looking at the cell cycle, we did not observe any differences in the frequency of cells at different cycle phases in the WT and HO-1 MIC^+^-derived cell lines when compared to their parental cell lines (Figure S4a). Next, we checked if MIC^+^-derived cell lines are enriched in cells expressing MIC markers. We found that the frequency of CD20^+^ cells was not significantly changed in progeny-derived cell lines, regardless of the founder cell phenotype (Figure 5b). The same was true for the frequency of CD133^+^ and CD24^+^ cells in the MIC^+^ progeny (Figure S4b). Surprisingly, ALDH^high^-derived cell lines showed a lower frequency of ALDH^high^ cells than their parental B16-F10 line, both in WT and HO-1-overexpressing cells (Figure 5c). Overall, we demonstrated that MIC^+^-derived cell lines were not enriched in MIC-expressing cells. 

Additionally, we analyzed the expression of MAAs. We compared CD20^−^- and CD20^+^-derived (Figure 5d) as well as ALDH^low^- and ALDH^high^-derived (Figure 5e) cell lines. We did not find any statistically significant differences in expression of *Tyr*, *Mitf*, *Gp100*, and *Mart-1*. Hence, cell lines derived from a single MIC^+^ cell can restore the heterogeneity of phenotype and gene expression profile typical for the parental cell line.

High activity of ALDH was previously described to be responsible for chemoresistance of different cancer cell types [54]. Therefore, we investigated if the progeny of ALDH^high^ cells is more resistant to doxorubicin. Cytotoxicity of doxorubicin was strongly pronounced in this set of experiments, but we did not observe any differences in response to the treatment between ALDH^high^ progeny-derived cell lines and control B16-F10 cells (Figure 5f). This result further confirms that the progeny of MIC^+^ cells reconstitute the features of the parental cell line.

### 2.6. CD20^+^ and ALDH^high^ Fractions Are Not Enriched in Tumorigenic Cells

The presence of CSCs in human melanoma has been put into question when it appeared that methodological changes in xenotransplantation assays seem to have a higher influence on the tumorigenic potential of melanoma cells than the expression of MIC markers [25,26]. As there is a need for further evaluation of the tumorigenic potential of melanoma cells in immunocompetent hosts, we used B16-F10 cells for syngeneic transplantations to C57BL/6 mice.

**Figure 6:**
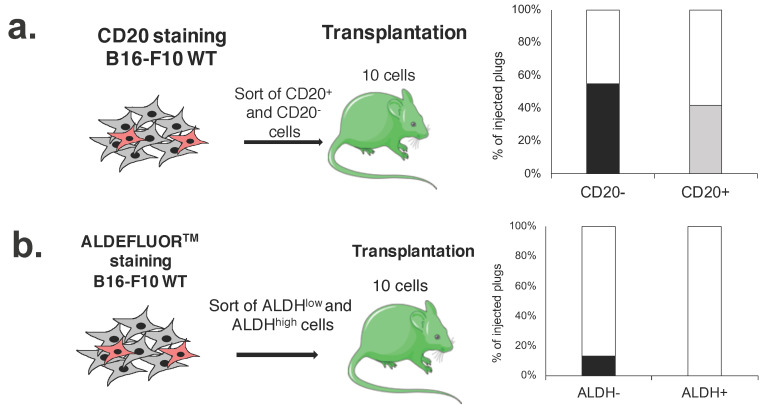
In vivo evaluation of tumorigenic potential of MIC subsets using syngeneic mice. Transplantation of 10 sorted cells for a chosen marker: (**a**) CD20^−/+^ and (**b**) ALDH^low/high^ (10 cells/plug, 2 plugs/mouse, n = 11–12 mice/group).

First, we performed in vivo transplantation of 10 sorted CD20^−^ and CD20^+^ cells per plug (two plugs per mouse) to the C57BL/6 syngeneic immunocompetent mice (Figure 6a). We observed that both fractions showed high tumorigenic efficacy and there was no difference in the formation of tumors between CD20^−^ and CD20^+^ cells (Figure 6a). Additionally, tumors formed by both CD20^−^ and CD20^+^ fractions had similar metastatic potential and resulted in similar survival outcome of mice (Figure S5). These results confirm our in vitro observations where CD20^−^ and CD20^+^ melanoma cells displayed similar clonogenicity after single-cell sorting (Figure 4b). We performed the same experiment using sorted ALDH^low^ and ALDH^high^ cells. Interestingly, cells with high ALDH activity were unable to form any tumors (Figure 6b).

### 2.7. Overexpression of HO-1 Enhances the Survival of Melanoma Cells but Decreases Their Self-Renewal and Tumorigenicity in Serial Transplantation Assay

**Figure 7:**
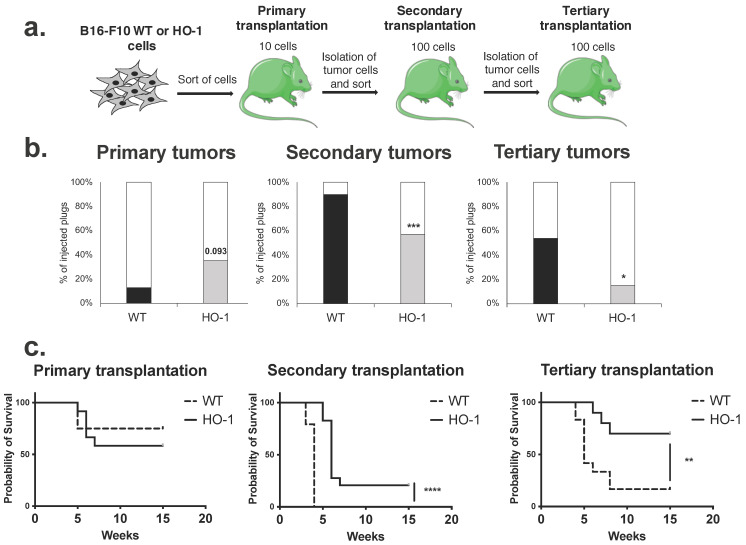
Serial transplantation of B16-F10 WT and HO-1 cells. (**a**) Experimental layout. Primary recipients (C57BL/6-Tg(UBC-GFP)30Scha/J mice) were injected with 10 sorted cells per Matrigel plug (2 plugs/mouse). After tumor formation, mice were sacrificed, tumors were excised, and GFP^−^/7AAD^−^/Hoechst^+^ cells were sorted and transplanted (100 cells/plug) to the secondary recipients. (**b**) Efficacy of tumor formation represented as % of injected plugs/mouse (primary recipients n = 12 mice/group, secondary recipients n = 29 mice/group, tertiary recipients n = 10–13 mice/group; Fisher exact test; * *p* < 0.05, *** *p* < 0.001 versus WT). (**c**) Survival curve of mice. Mice were sacrificed when tumors reached 10 mm in diameter (Mantel–Cox test; ** *p* < 0.01, **** *p* < 0.0001).

Our in vitro experiments suggested that HO-1 overexpression increases the expression of CSC-associated genes and some CSC features (Figure 1) but at the same time decreases the clonogenicity of B16-F10 melanoma cells (Figure 3). To ultimately verify the effect of HO-1 on melanoma tumorigenicity and self-renewal of the cells, we performed an in vivo serial transplantation assay, the gold standard in research on CSC tumorigenicity [55]. We sorted B16-F10 WT and HO-1 cells and injected 10 cells per plug to the primary recipients, namely to the GFP-expressing C57BL/6 transgenic immunocompetent mice (Figure 7a). After the formation of primary tumors, mice were sacrificed, tumors were digested, and 100 tumor-derived cells were injected into the secondary recipients. The same procedure was performed for the tertiary recipients (Figure 7a). The data showed that injection of B16-F10 WT melanoma cells resulted in the formation of tumors in 15% of injected plugs (Figure 7b). Overexpression of HO-1 increased the efficacy of primary tumor formation up to 35% (Figure 7b), which might be a result of improved melanoma cell survival [39]. Interestingly, when primary tumors were transplanted to the secondary recipients, up to 90% of transplanted plugs formed tumors in the WT group, but only 57% of transplanted plugs formed tumors in the HO-1 group. A similar relationship, with a lower tumor formation rate by HO-1-overexpressing cells, was found in tertiary transplantation (Figure 7b). As a consequence, we observed better survival of animals in secondary and tertiary recipients of HO-1 cells (Figure 7c). Finally, we did not observe any difference in the metastatic potential of WT and HO-1 cells (data not shown). Generally, these data indicate that overexpression of HO-1 decreases the self-renewal and tumorigenicity of melanoma cells in serial in vivo transplantation assay. This is in line with the decreased clonogenic potential of these cells observed in vitro. 

Taken together, our in vitro and in vivo data proved that HO-1 has a greater effect on melanoma clonogenic and tumorigenic potential than the expression of melanoma-initiating cell markers in the murine B16-F10 cell line. 

## 3. Discussion

Melanoma aggressiveness, heterogeneity, and plasticity pose a major challenge for the treatment of this cancer. Despite reports that identify MIC subsets in melanoma tumors [10,11,12,13,14,15,16,17,18,19,20,21,22,23,24], the presence of melanoma cells that follow the classical CSC hierarchical model has been put into question by many other studies [25,26,27,28,29]. Thus, the controversies around the existence, behavior, and factors that regulate MICs need to be further addressed and are a subject of this study. We focused on the characterization of MIC subsets in the B16-F10 murine melanoma cell line that enabled us to perform syngeneic in vivo cell transplantation assays.

Using flow cytometry analyses, we identified several subsets of cells expressing known CSC-associated markers. Previously, Kuch and colleagues checked several MIC markers in the B16-F10 cell line and found CD133^+^, ALDH^high^, and CD44^+^ populations but no expression of CD20, CD24, and CD34 [56]. In contrast, we were able to consistently identify small subsets of CD20^+^ and CD24^+^ in our experiments (Figure 2a,b). The expression of CD133 in the B16-F10 line seems to be consistent in several studies [56,57,58], including ours, and accounts for a very small subpopulation (0.15% on average). However, in human patient-derived melanoma cell lines, CD133 is heterogeneously expressed and ranges from 0% to more than 60% of positive cells [59].

Identification of cells with a high activity of ALDH was less straightforward. Initially, we identified quite a large subpopulation of ALDH^high^ cells (16.8% in normoxia and 38.4% in hypoxia), similar to other reports [56]. However, in some experiments, we observed a drastic decrease in the number of ALDH^high^ cells in the B16-F10 lines, despite identical experimental settings. This might be explained by the high sensitivity of ALDH^high^ cells to culture conditions such as oxygen supply (Figure 2d), non-adherent growth [56,58], and cell density [60]. Generally, we faced high variability of the percentage of MIC antigen expression between experiments. This can reflect the high plasticity of melanoma cells and has been observed before by other groups [61,62]. We believe that the B16-F10 line can serve as a model for studies on MIC and MIC markers. However, one should not focus on the averaging of absolute numerical values from different experiments but rather directly comparing MIC^+^ and MIC^−^ subsets from the same cell cultures.

In breast cancer, increased expression of HO-1 augments the aggressiveness of CD24^low^ CD44^high^ CSC fraction and marks mammospheres [63]. Recent data on human melanoma proved that HO-1 activity promotes the formation of melanospheres [64]. Consistently, we observed that inhibition of HO-1 activity with SnPP abolished non-adherent growth (Figure 1a) without changing the HO-1 expression (data not shown), which highlights the important role of enzymatic activity of heme oxygenase in the formation of melanospheres by murine melanoma. Moreover, HO-1 overexpression led to enhanced formation of tube-like structures on Matrigel by melanoma cells, the feature attributed to vasculogenic mimicry [50]. It was accompanied by the increased expression of many genes associated with increased VM, including *Notch1* [65], *Fzd7* [66], *Snai2*, and *Jak2* [67]. Altogether, we propose that HO-1 might influence VM through upregulation of CSC-associated pathways, but this needs further study.

Overexpression of HO-1 in human melanoma cells led to enhanced colony formation, and the opposite was observed when HO-1 was silenced [45]. The same dependencies were described in cervical carcinoma [68], thyroid cancer [69], leiomyomatosis and renal cell carcinoma [70], and human pancreatic cancer cells [71]. Unexpectedly, we observed that overexpression of HO-1 in B16-F10 melanoma cells leads to decreased clonogenicity both in normoxia and hypoxia and HO-1 clones were significantly smaller than their WT counterparts (Figure 3). To assess the influence of HO-1 on MIC subsets, we chose CD20 and ALDH cells for further analysis. Although CD20 was shown to be associated with increased melanosphere formation [72], to our knowledge the clonogenic capacity of CD20^+^ melanoma cells has not yet been characterized. We did not observe any significant differences in clonogenicity of CD20^−^ and CD20^+^ cells in either normoxia or hypoxia (Figure 4a), which was confirmed by our in vivo data (Figure 6a).

Knockdown of ALDH1A3 led to decreased clonogenicity in neuroblastoma [73] and non-small-cell lung carcinoma [74], whereas ALDH^high^ cells were associated with increased clonogenicity in Ewing’s sarcoma [75]. Again, we did not observe any significant differences in clonogenic potential in vitro between ALDH^low^ and ALDH^high^ subsets in WT control. Just like in unfractionated cells, overexpression of HO-1 decreased clonogenicity of melanoma, regardless of the MIC status. To sum up, we did not detect any significant influence of MIC expression on the clonogenicity of melanoma cells. Instead, our results indicate that it is HO-1 overexpression that primarily affects clonogenicity and morphology of melanoma clones, not MIC marker status.

One of the characteristics of CSCs is the ability to give rise to more differentiated progeny, which is reflected in the heterogeneity of cancers [6]. The true CSC^+^ should be able to give rise to both CSC^−^ and CSC^+^ progeny due to the self-renewal and differentiation abilities. At the same time, in the unidirectional model of CSC, CSC^−^ fraction should not be able to give rise to CSC^+^ population [76]. However, there is a growing body of evidence showing that the plasticity of CSC progeny can lead to the formation of CSC^+^ from non-CSC fractions [76]. Examination of MIC progeny in our study revealed that they re-establish heterogeneity of parental cells in terms of expression of MIC markers and expression of MAAs (Figure 5). Interestingly, ALDH^high^ derived cell lines had reduced ALDH^high^ fraction when compared to the parental cell lines. It should be stressed once again, however, that we observed particular heterogeneity in melanoma ALDH activity.

We did not find any influence of HO-1 overexpression on phenotypic heterogeneity of melanoma. Our results are in line with the report published by Huang and colleagues showing that, over time, sorted and cultured CSC^+^ and CSC^−^ cells (in different types of cancers) regained the proportions of CSC^+^ to CSC^−^ cells characteristic of the parental cell lines [77]. The same authors also pointed at an important issue—that CSC^−^ and CSC^+^ cells do not differ in terms of Ki67^+^ proliferating cells, suggesting that expression of CSC markers is not necessarily connected with a quiescent phenotype [77]. We also did not observe any differences in the cell cycle of MIC progeny, both in WT and HO-1 cell lines (Figure S4a). In vivo studies in human melanoma proved that different subsets of cells expressing CSC markers (e.g., CD271, ABCB5) were able to recapitulate the heterogeneity of parental tumors when injected into NSG mice [26]. Thus, the phenomenon of re-establishment of heterogeneity after purification of different subsets of melanoma cells is consistent in human and mouse cancers and seems to be irrespective of a marker used.

In the last part of our study, we characterized the tumorigenicity of cells expressing MIC markers. Targeting CD20 with engineered cytotoxic lymphocytes (CTLs) causes the elimination of human melanoma biopsies transplanted to immunocompromised mice [78]. This was observed even though the CD20 fraction was calculated to constitute only 2% of all tumor cells, meaning that targeting of small subpopulations of cells can lead to the elimination of not only CD20^+^ cells but also bulk tumor cells [78]. Moreover, the administration of anti-CD20 antibody rituximab in late-stage melanoma patients caused long-lasting remission of injected melanoma lesions and increased overall survival [79,80]. These data indicate that the CD20^+^ fraction plays an important role in human melanoma development. However, in our experiments, we did not observe any differences in tumorigenic potential between CD20^−^ and CD20^+^ fractions in syngeneic cell transplantation (Figure 6a). Overall, our data suggest that expression of CD20 does not mark cells with MIC properties in a murine melanoma cell line both in vitro and in vivo.

Similarly, we found that the high activity of ALDH does not mark MIC subsets in murine melanoma. Some previous reports showed that in human melanoma ALDH^high^ cells are highly tumorigenic when compared to control ALDH^low^ cells [20,81]. However, one study by Prasmickaite and colleagues did not find enhanced tumorigenicity and CSC properties within the ALDH^high^ fraction, in accordance with our study [29]. What connects our experiments with those described by Prasmickaite is the use of immortalized cell lines to investigate CSCs, which can influence tumorigenicity. In studies where the ALDH^high^ fraction was described as a population with CSC properties, the researchers used freshly obtained samples from melanoma patients [20,81]. Interestingly, one study on the B16-F10 murine melanoma identified the ALDH^high^ fraction as less tumorigenic than ALDH^low^ cells [56]. This is consistent with our experiments, where only the ALDH^low^ and not the ALDH^high^ fraction was able to initiate tumor growth after transplantation of 10 cells (Figure 6b). Such an effect might be related to the decreased expression of several CSC genes such as *Lats1*, *Stat3*, and *Foxp1* in the ALDH^high^ cells (data not shown) reported to be associated with melanoma aggressiveness [82,83,84]. However, the significance of these genes in the potentially reduced tumorigenicity of ALDH^high^ B16-F10 cells requires experimental verification.

Our previous study showed that although overexpression of HO-1 in melanoma cells did not affect the size of growing tumors after subcutaneous transplantation to syngeneic mice; the density of melanoma cells within the tumors overexpressing HO-1 was higher than that in the wild-type control [39]. Accordingly, overexpression of HO-1 in a human melanoma cell line caused increased tumor growth [45]. However in both studies, the number of transplanted cells was high, so it was proliferation rate and survival of injected cells that were measured, not initiation of tumor growth. Our data suggest that a high level of HO-1 in single melanoma cells is not favorable for the initiation of clonal proliferation in vitro. To further investigate the effect of HO-1 on tumor initiation capacity, we performed in vivo serial transplantation of WT and HO-1-overexpressing cells into syngeneic C57BL/6J mice. According to the CSC theory, injection of a very small number of CSCs, but not bulk cells, into mice should result in tumor formation [85]. In our experimental setting, injection of only 10 WT or HO-1-overexpressing B16-F10 melanoma cells resulted in the formation of tumors. HO-1 increased tumorigenicity in primary recipients but significantly decreased initiation of tumors in secondary and tertiary recipients (Figure 7). Therefore, we can interpret the data in the following two ways: First, B16-F10 cells can initiate tumor growth from a very limited number of cells when transplanted to the syngeneic recipients, similar to what was observed in human melanoma [25]. Human melanomas were highly tumorigenic even when single cells were injected into NSG mice—this result put into question the existence of CSCs in melanoma [25]. Here, we showed that murine melanoma cells transplanted to immunocompetent, syngeneic mice also have a pronounced tumorigenic potential. Second, we showed that overexpression of HO-1 decreases tumorigenicity in secondary and tertiary recipients, probably due to reduced self-renewal of melanoma cells. Early induction of HO-1 in Mdr2^−/−^ mice (the model of chronic liver inflammation and inflammation-induced tumor development) delayed the initiation of liver tumors through amelioration of chronic inflammation [86]. Moreover, in chemically induced squamous cell carcinoma, HO-1 KO mice developed lesions earlier than WT animals [87], suggesting that HO-1 can delay the initiation of tumor formation. Here we found that in B16-F10 melanoma, HO-1 increased the proliferation of MICs in primary recipients but reduced the induction of secondary and tertiary tumors. Overall, HO-1 probably improves the survival of transplanted cells but decreases initiation of tumor growth in the cell self-renewal-dependent long-term transplantation assay. This is consistent with diminished clonogenicity observed in vitro.

In summary, we conclude that the expression of MIC markers does not select CSC-like cells in a murine melanoma cell line. MIC^−^ and MIC^+^ subsets display similar clonogenicity in vitro and tumorigenicity in vivo, and progeny of both MIC^−^ and MIC^+^ cells regain heterogeneity of the bulk subpopulation. This supports the view that melanoma does not follow the classical CSC model and that melanoma cells with tumor initiation capabilities are not rare. Moreover, HO-1 has a dual role in melanoma. As a previous study of our group showed that overexpression of HO-1 increased aggressiveness of bulk B16-F10 cells in growing tumors [39], here we found that HO-1 might decrease the risk of melanoma initiation. We demonstrated that overexpression of HO-1 during clonal growth induction in vitro and in vivo can play an antitumorigenic role. Our results draw a broader picture of melanoma therapy and suggest that pharmacological inhibitors of HO-1 in melanoma treatment might have a different effect on tumor growth than on tumor initiation.

## 4. Materials and Methods

### 4.1. Cell Culture

B16-F10 murine melanoma cell line (ATCC, Manassas, VA, USA) was routinely cultured in RPMI 1640 medium supplemented with 2 mM L-glutamine (Lonza, Basel, Switzerland), 10% inactivated fetal bovine serum (FBS, Eurx, Gdansk, Poland), and 10,000 units/mL of penicillin and 10 mg/mL streptomycin (PEST, Sigma-Aldrich, Saint Louis, MO, USA). Cells were cultured in standard conditions (5% CO_2_, 37 °C, 95% humidity) and passaged every 2–3 days. For clonogenic assays and melanosphere formation, cells were cultured in melanoma-initiating cell (MIC) medium containing DMEM/F12 (Lonza, Basel, Switzerland), PEST, 0.6% glucose (Sigma-Aldrich, Saint Louis, MO, USA), 1x supplement N_2_ (Life Technologies, Santa Clara, CA, USA), 20 μg/mL human recombinant insulin (Sigma-Aldrich, Saint Louis, MO, USA), 10 ng/mL basic fibroblast growth factor (bFGF, PeproTech, London, UK), and 10 ng/mL epidermal growth factor (EGF, PeproTech, London, UK) [48]. Hypoxic cultures were performed using a hypoxia chamber (0.5% O_2_, NuAire chamber or Don Whitley hypoxic chamber from Bentley Polska).

Human embryonic kidney cells 293 (HEK293) were kindly gifted by Dr. Maciej Wiznerowicz (Greater Poland Cancer Center, Poznan, Poland) and used for lentiviral production. Cells were cultured in DMEM HG supplemented with 10% FBS and PEST in standard conditions (5% CO_2_, 37 °C, 95% humidity).

Phoenix Ampho cells (ATCC, Manassas, VA, USA) were used as a packaging cell line for retroviral production and were cultured routinely in DMEM HG supplemented with 10% FBS and PEST in standard conditions (5% CO_2_, 37 °C, 95% humidity).

### 4.2. Generation of Stable B16-F10 Luc HO-1 Cell Line

Stable expression of the firefly luciferase gene (Luc) was obtained by the lentiviral transduction of B16-F10 cells. Briefly, lentiviruses (LVs) were produced in HEK293 cells transfected using polyethyleneimine (*M*_W_, Polysciences Inc., Warrington, PA, USA) with plasmids pMD2.G, psPAX2, and pLenti PGK V5-Luc Neo (all from Addgene, Watertown, NY, USA). Medium with LVs was collected 48 h after transfection, filtered (0.45 μm PVDF filter), and used for infection of B16-F10 cells. After 48 h, successfully transduced cells were selected in RPMI CM supplemented with 0.8 mg/mL G418 (CytoGen GmbH, Wetzlar, Germany).

For stable HO-1 overexpression, B16-F10 Luc cells were transduced with retroviral vectors (RVs) harboring HO-1 transgene. RVs were produced in Phoenix-Ampho transfected using polyethyleneimine (*M*_W_, Polysciences Inc., Warrington, PA, USA) with packaging plasmid M13 and pBABE-Puro-HO-1 plasmid (HO-1 transgene cloned into the pBABE-Puro plasmid from Addgene). Transduced B16-F10 HO-1 Luc cells were selected using 2 μg/mL puromycin (Sigma-Aldrich, Saint Louis, MO, USA). In some experiments, we used the B16-F10 HO-1 GFP-Luc cell line that was generated in our previous study [46].

### 4.3. Animals

In vivo studies were performed on C57BL/6-Tg(UBC-GFP)30Scha/J mice (obtained from The Jackson Laboratory, Bar Harbor, FL, USA). Mice were bred in a specific-pathogen-free (SPF) animal facility at the Faculty of Biochemistry, Biophysics and Biotechnology of Jagiellonian University. Mice were kept in individually ventilated cages (IVCs) and were regularly monitored according to Federation of European Laboratory Animal Science Association (FELASA) recommendations. All procedures were approved by the Local Institutional Animal Care and Use Committee (approval number 139/2015).

### 4.4. Detection of Cell Surface Markers Using Flow Cytometry

To recognize co-expression of MIC markers, B16-F10 cells were stained for several antigens simultaneously, and fluorescence minus one (FMO) samples were used as controls. After 48 h of culture in normoxia (21% O_2_) or hypoxia (0.5% O_2_), 1 × 10^6^ cells were stained in 100 μL of staining buffer (phosphate-buffered saline (PBS) without Ca^2+^ and Mg^2+^ (Lonza, Basel, Switzerland) + 2% FBS + 0.2 μg/mL DAPI (Sigma-Aldrich, Saint Louis, MO, USA). Stainings were performed with antibodies listed in Table 1. Detection of ABCB1 and ABCB5 required staining with primary and secondary antibodies. The analysis was performed using BD LSR II (BD Bioscience, Franklin Lakes, NJ, USA) flow cytometer.

### 4.5. ALDH Activity Assay

ALDH activity was measured using the ALDEFLUOR kit (StemCell Technologies, Vancouver, BC, Canada). Staining of 0.5–1.0 × 10^6^ cells was performed according to the vendor’s protocol, and samples were incubated for 30 min at 37 °C. Cells were additionally stained with DAPI for viability assessment. ALDH activity was analyzed using BD LSR II or BD Fortessa (BD Bioscience, Franklin Lakes, NJ, USA) flow cytometer.

### 4.6. Quantitative RT Polymerase Chain Reaction (qRT-PCR)

RNA isolation was performed by phenol–chloroform extraction as described previously [88]. The quality and concentration of isolated RNA were measured with a Nanodrop ND-1000 spectrophotometer. Synthesis of cDNA from total RNA was performed using the RevertAid First Strand cDNA Synthesis Kit (Thermo Scientific, Waltham, MA, USA) and qRT-PCR was performed using the SYBR Green JumpStart Taq ReadyMix (Sigma-Aldrich, Saint Louis, MO, USA) according to vendors’ protocols. qRT-PCR was performed using StepOnePlus thermocycler (Applied Biosystems, Waltham, MA, USA). Sequences and melting temperatures of primers are included in Table 2.

### 4.7. AmpliGrid Pre-Amplification System for Gene Expression Analysis from a Limited Number of Cells

Cells were sorted (50 cells/reaction site) based on their MIC phenotype on AmpliGrid (Munich, Germany) slides. After the microscopic evaluation of sorted cells, slides were kept at 4 °C overnight to dry. The RT was performed using the NCode Vilo mRNA cDNA Synthesis Kit (Invitrogen, Waltham, MA, USA) according to the vendor’s protocol in Stratagene Mx3005P cycler (Agilent, Santa Clara, CA, USA). The reaction was scaled down to 1 μL of reaction mix/reaction site. Five microliters of the cover oil was pipetted to each reaction site to prevent the evaporation of samples during PCR. After RT, 4 μL of H_2_O was carefully pipetted through the oil to dilute the cDNA sample, and samples were collected in separate tubes and used for qRT-PCR as described above.

### 4.8. RT^2^ Profiler PCR Array

For the RT^2^ Profiler PCR Array analysis, we sorted 2000–10,000 MIC^+^ and MIC^−^ B16-F10 cells in 100 μL of Buffer RL (Norgen, Thorold, Canada). Next, RNA was isolated using the Single Cell RNA Purification Kit (Norgen, Thorold, Canada) with the On-Column DNA Removal (Norgen, Thorold, ON, Canada) step according to the vendor’s protocol. RNA was eluted using 10 μL of DNase RNase-free H_2_O (this step was repeated 4 times to increase the RNA yield). Isolated RNA was entirely used for the RT-PCR using NCode Vilo (Invitrogen, Waltham, MA, USA), and obtained cDNA was diluted 6 times. The RT^2^ Profiler PCR Array (Qiagen, Germantown, MD, USA) detecting murine CSC-related genes was performed according to the vendor’s protocol using the Applied Biosystems StepOne Plus (Waltham, MA, USA) device. The list of genes is included in Table S1.

### 4.9. In Vitro Matrigel-Based Tube Formation Assay

B16-F10 WT Luc and B16 HO-1 GFP Luc cells were seeded (25,000/well) on 96-well plates coated with Matrigel GFR (50 µL of Matrigel/well, Corning Inc., Corning, NY, USA). Pictures were taken 3 and 20 h after seeding.

### 4.10. MTT Assay

Cells were seeded on the 96-well plates (1500 cells/well, in triplicates) in RPMI CM. The next day, cells were treated with different concentrations of doxorubicin (Sigma-Aldrich) for 24 h. The next day, cells were incubated for 20 min at 37 °C in RPMI supplemented with 1 mg/mL thiazolyl blue tetrazolium bromide (MTT, Sigma-Aldrich) and then lysed with the lysis buffer (containing 10 g of sodium dodecyl sulfate (SDS) and 0.6 mL of 100% acetic acid in 100 mL of DMSO). Absorbance was read at 562 nm using the Tecan Infinite M200 Pro Reader (Mannedorf, Switzerland).

### 4.11. Clonogenicity Test and Obtaining Cell Lines from Single Clones

Cells were stained with a proper antibody (see Table 1), and single cells were sorted using the MoFlo XDP (Becton Dickinson, Franklin Lakes, NJ, USA) on the 96-well plates. Cells were cultured in the MIC medium. Pictures of clones were taken every second day, starting from day 5. After two weeks, wells with clones were regarded as positive events. Cell lines derived from single clones were further cultured in the MIC medium and used for other assays.

### 4.12. Soft Agar Assay

Soft agar assay was performed in the 2× RPMI: 1.4 g of RPMI powder; 0.2 g of sodium bicarbonate (both from Sigma-Aldrich, Saint Louis, MO, USA) and 50 mL of H_2_O with pH adjusted to 7.8–8.4 pH using pH meter. The medium was supplemented with 20% FBS, 2× PEST, and 2% Glutamax (ThermoFisher, Waltham, MA, USA) and filtered using a 0.2 μm filter (Millipore, Burlington, MA, USA). The bottom layer (0.8%) of the soft agar was prepared by adding an agar solution to the 2× RPMI CM in a 1:1 ratio. Next, 400 μL of the solution was quickly added to pre-heated 12-well culture dishes. The proper numbers of cells (final density of 2000 cells/well) were mixed with the 2× RPMI CM, and then 0.8% agar was added in a 1:1 ratio. Finally, 600 μL of agar with cells was added to each well as a middle layer. After 30 min, the upper layer of 0.8% agar was added as described above. Cells were cultured for 7 days and colonies were counted under the microscope (6 wells per cell line and 10 fields of view per well were counted by two independent investigators).

### 4.13. In Vivo Injection of Cells

In vivo primary transplantation of cells was performed after staining of cells with CD20 antibody or ALDEFLUOR Kit (StemCell Technologies, Vancouver, Canada). Additionally, 0.2 μg/mL DAPI was used to distinguish live/dead cells. For each mouse, 30 cells were sorted into a 1.5 mL Eppendorf tube containing 150 μL of PBS. After the sorting, 150 μL of Matrigel GFR (Corning Inc., Corning, NY, USA) was added to each tube to obtain the concentration of 10 cells per 100 μL solution for injection. C57BL/6-Tg(UBC-GFP)30Scha/J mice were subcutaneously injected (2 plugs/mouse) with 100 μL of cell suspension under isoflurane (Baxter, Deerfield, FL, USA) anesthesia.

### 4.14. In Vivo Serial Transplantations

Primary injection of unfractionated B16-F10 WT Luc and B16-F10 HO-1 GFP Luc cells was performed in the same way as described above. When the primary tumors grew up to 1 cm in diameter, mice were euthanized with the use of CO_2_. Excised tumors were chopped with a scalpel and digested for 1 h at 37 °C in 2 mL of enzyme mix (containing 3 U/mL liberase, 25 μg/mL hyaluronidase, 25 μg/mL DNAse, and 3 U/mL dispase, all from Sigma-Aldrich, Saint Louis, MO, USA) as described by Szade et al. [89]. Digestion was stopped with RPMI 10% FBS, and the digested tissues were thoroughly pipetted and filtered using the 100 μm strainer. After washing with PBS, cells were centrifuged (600*g*, 5 min, room temperature) and pellets were stained in 500 μL PBS with 7-amino-actinomycin D (7AAD, diluted 20x, BD Pharmingen, San Diego, CA, USA) and Hoechst 33342 (160 μg/mL, Sigma-Aldrich, Saint Louis, MO, USA) (15 min, room temperature, in the dark). GFP^−^7AAD^−^Hoechst^+^ cells were sorted into PBS and diluted 1:1 with Matrigel to the final concentration of 100 cells/100 μL, and mice were subcutaneously injected (2 plugs/mouse) with 100 μL of solution (containing 100 cells) per plug (2 plugs/mouse). In the case of the B16-F10 HO-1 GFP Luc cell line, there was a GFP^−/dim^ subpopulation, additionally distinguished in the SSC/FSC plot from the host cells. Each primary tumor was transplanted to 9–10 C57BL/6-Tg(UBC-GFP)30Scha/J secondary recipients. Tertiary transplantations were performed similarly, but one secondary tumor was transplanted to one tertiary recipient.

### 4.15. Statistical Analysis

Data analysis was performed using the Prism 8 for Mac OS (GraphPad Software, San Diego, CA, USA) or Microsoft Excel for Mac (Microsoft Office 365, Excel version 16) software. Results are represented as mean + SEM, and each experiment was performed at least in two independent biological repetitions. Data were analyzed with two-tailed Student t-test (two groups), one-way or two-way ANOVA with Bonferroni post-test (three or more groups), and two-tailed Fisher exact test (for clonogenic events calculations and in vivo tumor formation). When comparing two groups with non-normal distribution, Mann–Whitney test was used (box and whisker plots). In vivo survival of mice was calculated using the Mantel–Cox test. Results were considered statistically significant at *p*-value < 0.05.

## Figures and Tables

**Table 1:** Antibodies used for flow cytometry analysis.

Antibody	Fluorochrome, Clone, Company	Dilution
ABCB1	Mouse monoclonal [JSB-1] to p-glycoprotein, Abcam (Cambridge, UK)	1:100
ABCB5	Rabbit polyclonal, Bioss (Woburn, MA, USA)	1:100
Sca-1	PE/Cy7 Anti-mouse Ly6A/E, clone E13-161.7 BioLegend (San Diego, CA, USA)	1:100
CD20	PE anti-mouse CD20, clone SA275A11, BioLegend (San Diego, CA, USA)	1:100
CD133	APC anti-mouse CD133, clone 315-2C11, BioLegend (San Diego, CA, USA)	1:100
CD24	PerCP-Cy5.5 Rat Anti-Mouse CD24, clone M1/69, BD Pharminogen (San Diego, CA, USA)	1:100
Goat anti-rabbit IgG	Alexa Fluor 488, A-11008, Invitrogen (Waltham, MA, USA) (ABCB5 staining)	1:100
Goat anti-mouse IgG	Alexa Fluor 488, A28175, Invitrogen (Waltham, MA, USA) (ABCB1 staining)	1:100

**Table 2:** Sequences of primers used in the study.

Primer	Sequence	Length of Product	Tm
*Ef2* For	5′ GACATCACCAAGGGTGTGCAG 3′	214 bp	60 °C
*Ef2* Rev	5′ TCAGCACACTGGCATAGAGGC 3′		
*Tyr* For	5′ GCCCAGCATCCTTCTTCTCC 3′	101 bp	55 °C
*Tyr* Rev	5′ TAGTGGTCCCTCAGGTGTTC 3′		
*Gp100* For	5′ ACCACTATGGGTGTCCAGAGA 3′	108 bp	60 °C
*Gp100* Rev	5′ GACACCAAGCCAGTCCTGAT 3′		
*Mitf* For *Mitf* Rev	5′ AGAGCAGGGCAGAGAGTGAGT 3′ 5′ CAGGAGTTGCTGATGGTAAGG 3′	238 bp	65 °C
*Hmox1* For *Hmox1* Rev	5′ GTGGAGACGCTTTACATAGTGC 3′ 5′ CTTTCAGAAGGGTCAGGTGTCC 3′	250 bp	60 °C
*Mart-1* For *Mart-1* Rev	5′ CAGTACCAGCAGCCGATAAGCA 3′	166 bp	55 °C
	5′ GGGAAGGTGTCCTGTGCTGAGT 3′		

## Data Availability

All data in the manuscript are available from the corresponding author upon request.

## Supplementary Materials

The following supporting information can be downloaded at: https://www.mdpi.com/article/10.3390/ijms23073596/s1. Refs. [90,91] are cited in supplementary materials.
