# Supplementary material for: Heme Oxygenase-1 Has a Greater Effect on Melanoma Stem Cell Properties Than the Expression of Melanoma-Initiating Cell Markers
Source: Int J Mol Sci. 2022 Mar 25;23(7):3596. doi: 10.3390/ijms23073596 (PMC8998882; doi:10.3390/ijms23073596)
Supplement: Supplementary file 1 [file ijms-23-03596-s001.zip › Figure S1.pdf]

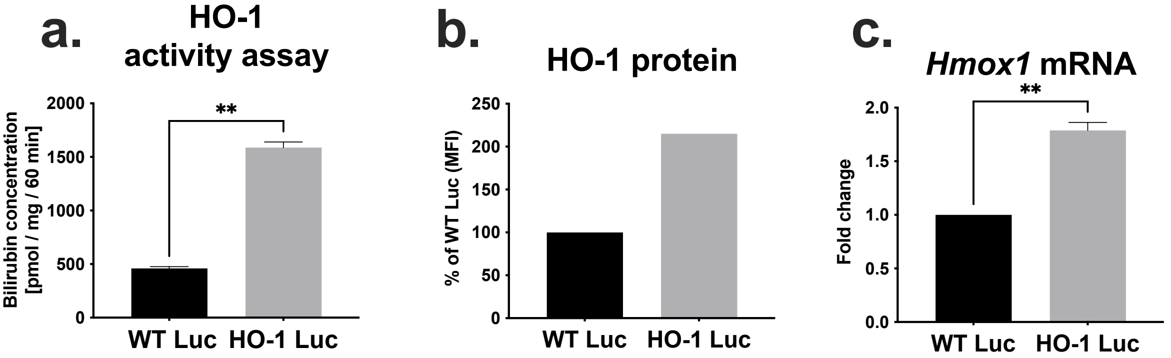

Figure S1. Characterization of B16-F10 HO-1 Luc cell line. (**a**) Measurement of HO-1 activity (bilirubin production assay). Data presented as mean+SD; ** p<0.01); **(b)** Detection of HO-1 protein (representative FACS analysis); **(c)** Detection of HO-1 mRNA (qRT-PCR)
