# Supplementary material for: Heme Oxygenase-1 Has a Greater Effect on Melanoma Stem Cell Properties Than the Expression of Melanoma-Initiating Cell Markers
Source: Int J Mol Sci. 2022 Mar 25;23(7):3596. doi: 10.3390/ijms23073596 (PMC8998882; doi:10.3390/ijms23073596)
Supplement: Supplementary file 1 [file ijms-23-03596-s001.zip › Figure S2.pdf]

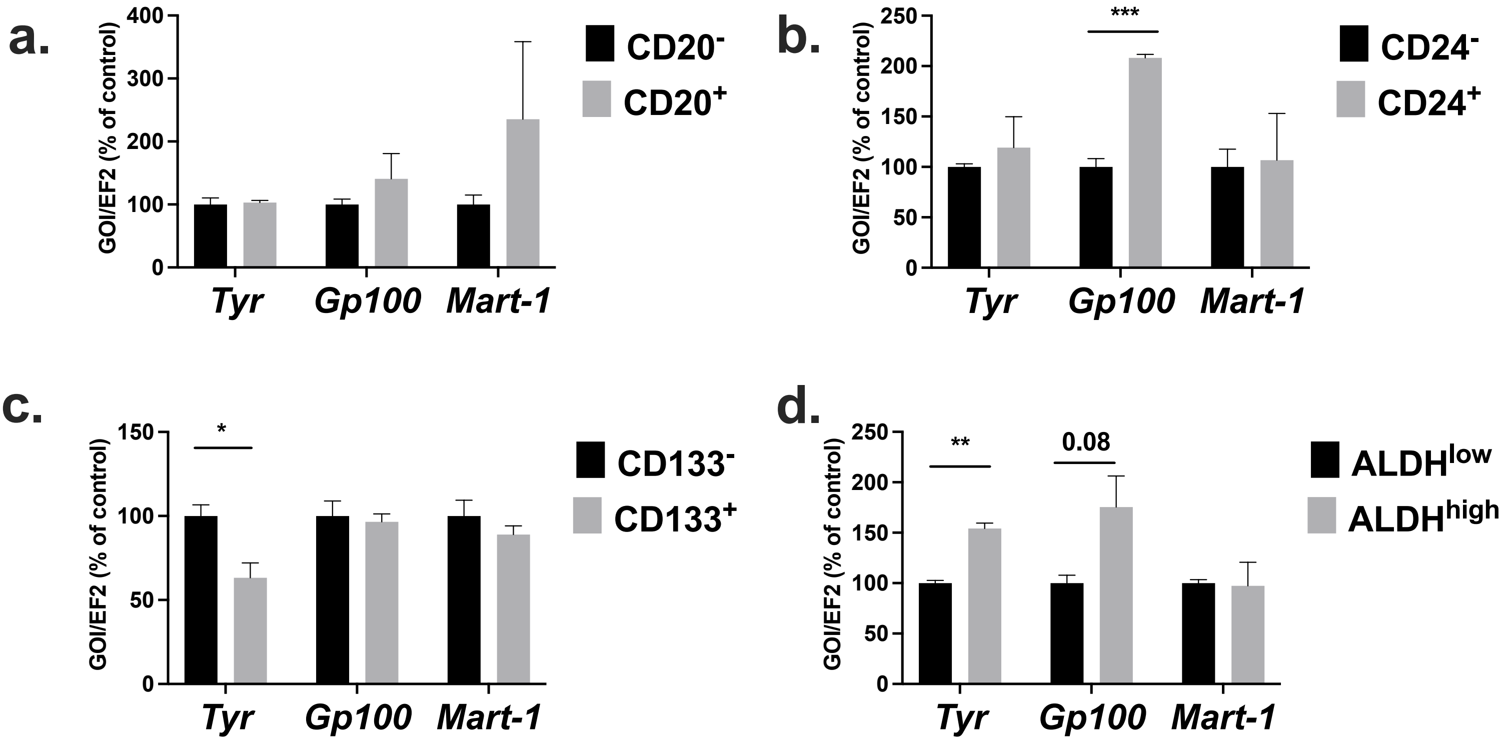

Figure S2. Expression of MAAs in MIC$^-$ and MIC$^+$ subsets. qRT-PCR analysis on 50 sorted cells **(a)** CD20$^{-/+}$, **(b)** CD24$^{-/+}$, **(c)** CD133$^{-/+}$ and **(d)** ALDH$^{low/high}$ cells, after pre-amplification with the AmpliGrid system; GOI – gene of interest; EF2 was used as a reference gene; each bar represents mean + SEM; * - p<0.05, *** - p<0.001 versus MIC- cells.
