# Supplementary material for: Heme Oxygenase-1 Has a Greater Effect on Melanoma Stem Cell Properties Than the Expression of Melanoma-Initiating Cell Markers
Source: Int J Mol Sci. 2022 Mar 25;23(7):3596. doi: 10.3390/ijms23073596 (PMC8998882; doi:10.3390/ijms23073596)
Supplement: Supplementary file 1 [file ijms-23-03596-s001.zip › Figure S3.pdf]

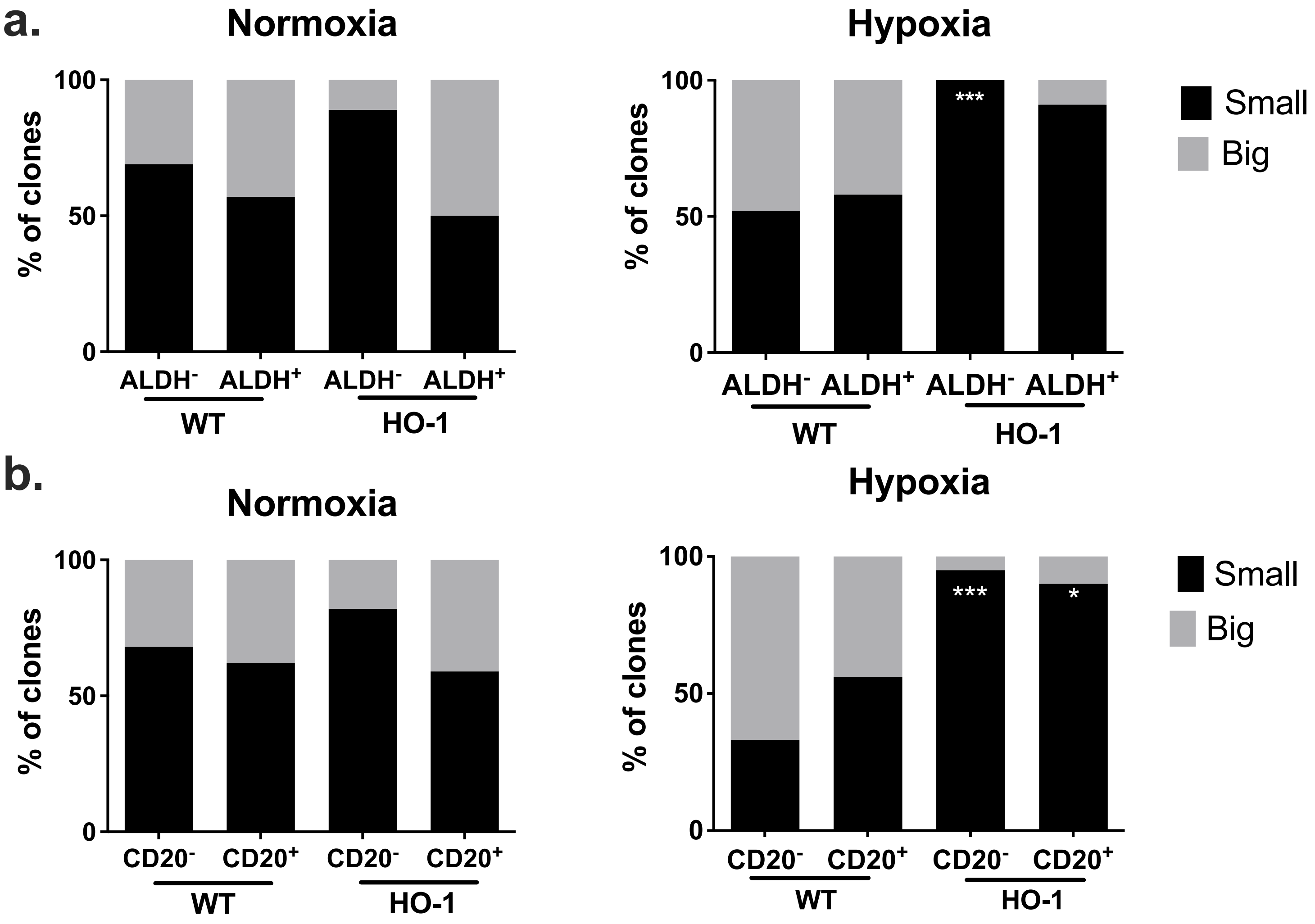

Figure S3. Morphology of MIC- and MIC+-derived clones 13 days after sorting. **(a)** Frequency of small and big ALDH^{high} and ALDH^{low} clones (n=9-32, Fisher's exact test); **(b)** Frequency of small and big CD20- and CD20+ clones (n=9-42, Fisher exact test; * - p<0.05, *** - p<0.001 versus WT).
