# Supplementary material for: Heme Oxygenase-1 Has a Greater Effect on Melanoma Stem Cell Properties Than the Expression of Melanoma-Initiating Cell Markers
Source: Int J Mol Sci. 2022 Mar 25;23(7):3596. doi: 10.3390/ijms23073596 (PMC8998882; doi:10.3390/ijms23073596)
Supplement: Supplementary file 1 [file ijms-23-03596-s001.zip › Figure S4.pdf]

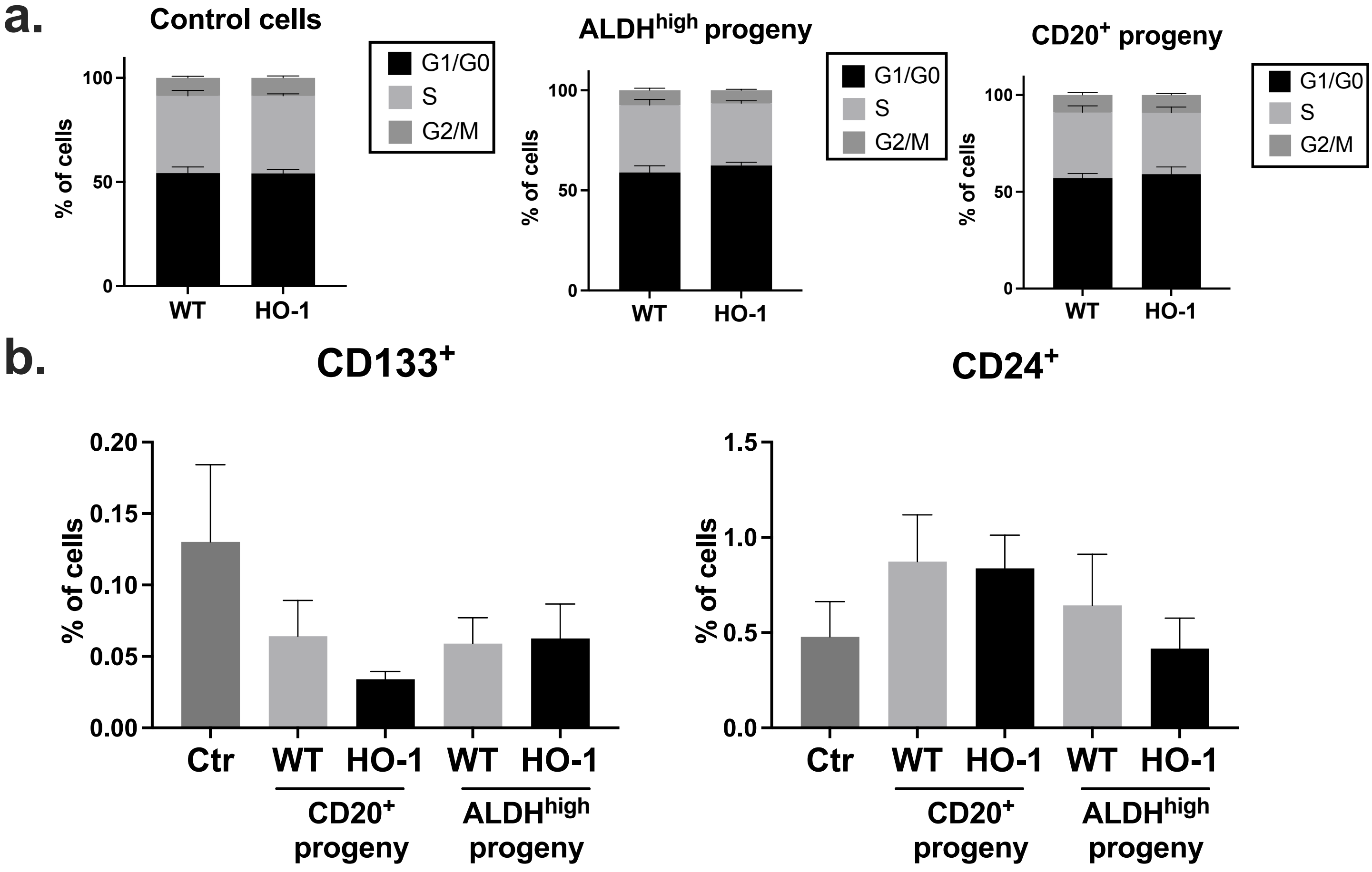

Figure S4. Flow cytometry phenotyping of MIC progeny. **(a)** Frequency of cells at different cell cycle phases in parental (control) cell lines and in cell lines derived from ALDH^{high} and CD20^{+} clones, bars represent means + SEM; **(b)** Frequency of CD133^{+} and CD24^{+} cells in CD20+ and ALDH^{high}-derived clonogenic cell lines and in the control cell line (WT Luc). Flow cytometry analysis, each bar represents mean + SEM.
