# Supplementary material for: Heme Oxygenase-1 Has a Greater Effect on Melanoma Stem Cell Properties Than the Expression of Melanoma-Initiating Cell Markers
Source: Int J Mol Sci. 2022 Mar 25;23(7):3596. doi: 10.3390/ijms23073596 (PMC8998882; doi:10.3390/ijms23073596)
Supplement: Supplementary file 1 [file ijms-23-03596-s001.zip › Figure S5.pdf]

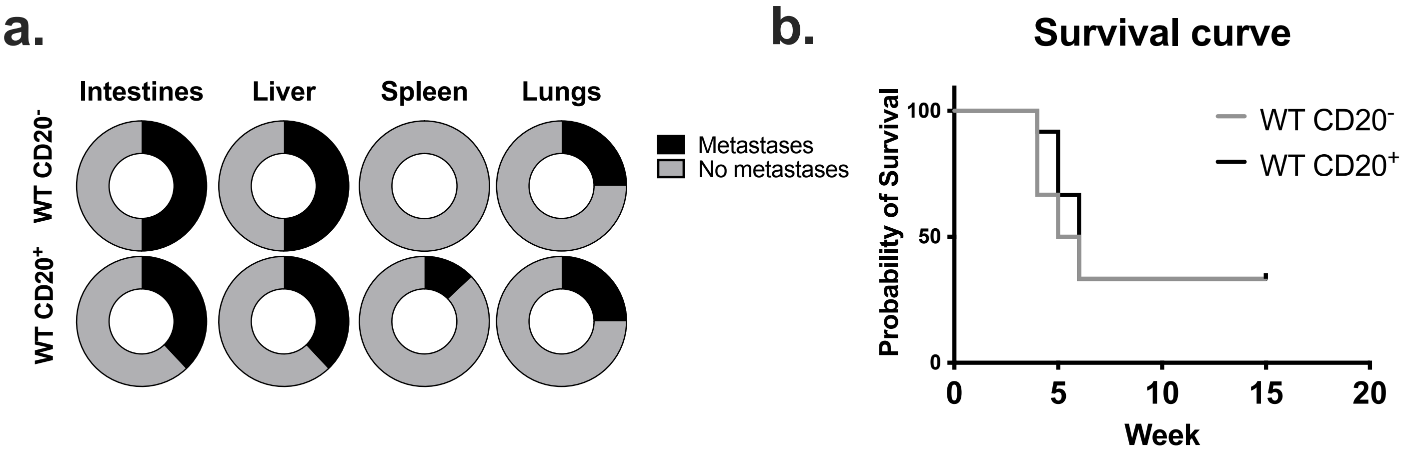

Figure S5. Metastases formation and survival of C57BL/6 mice injected with $CD20^-$ and $CD20^+$ cells. **(a)** Frequency of metastases measured by detection of luciferase activity in the *post-mortem* excised organs using IVIS luminometer (n=10-12). **(b)** Survival curves of $CD20^-$ and $CD20^+$ recipients. Mice were sacrificed when tumors reached 10 mm in diameter.
