# Supplementary material for: Heme Oxygenase-1 Has a Greater Effect on Melanoma Stem Cell Properties Than the Expression of Melanoma-Initiating Cell Markers
Source: Int J Mol Sci. 2022 Mar 25;23(7):3596. doi: 10.3390/ijms23073596 (PMC8998882; doi:10.3390/ijms23073596)
Supplement: Supplementary file 1 [file ijms-23-03596-s001.zip › Supplementary Materials and Methods.pdf]

International Journal of *Molecular Sciences*

Supplementary Materials and Methods

Cell cycle analysis

For cell cycle analysis cells were fixed with 70% ice-cold ethyl alcohol (EtOH, POCh) and incubated for at least 3h in -20°C. Then, cells were centrifuged (4200 *g*, 10 minutes) and stained with 250 ul of PBS containing propidium iodide (PI, Sigma-Aldrich, 1:100) and RNAse A (1:1000, Merck) for 30 minutes in the room temperature, in the dark. Samples were harvested using BD LSR II (BD Bioscience) flow cytometer and analyzed with ModFit software.

Post mortem detection of metastases

On the day of ending *in vivo* experiment, when tumors reached around 1 cm in diameter, mice were injected i.p. with 150 µl of the luciferin (15 mg/ml, TriMen Chemicals). After 20 minutes, mice were euthanized, organs were excised, and luminescence signals were measured with IVIS Lumina (PerkinElmer).

Heme oxygenase activity test

Heme degradation leads to the formation of biliverdin that is further metabolized by the biliverdin reductase (BVR) to bilirubin. The concentration of the latter can be measured spectrophotometrically in the heme oxygenase activity test. Test was performed according to the protocol described previously by Foresti et al[1]. Briefly, the reaction mixture contained: cell lysate (obtained by freezing/thawing technique using dry ice), BVR (recombined form, described by Mucha et al.[2] or the rat's liver lysate), NADPH (0.8 mM, Sigma-Aldrich), glucose-6-phosphate (2 mM, Sigma-Aldrich), glucose-6-phosphate dehydrogenase (0.2 units, Sigma-Aldrich), PBS containing 2mM $MgCl_2$ (0.2 mM, Sigma-Aldrich) and hemin (20 µM; Sigma-Aldrich). Samples were incubated for 1 h at 37 ºC in the dark. The reaction was stopped by the addition of 1 ml of chloroform and vigorous vortexing. Samples were centrifuged (500 *g*, 5 minutes) and a bottom organic phase containing reaction products was used for absorbance measurement at 464 nm and 530 nm using Tecan Infinite® 200 PRO spectrophotometer and quartz cuvette. The heme oxygenase activity was calculated using the formula:

$$\frac{A_{464}-A_{530}}{40\times(protein\ content)}\times 10^6\left[\frac{pmol}{mg/60\ min}\right],$$

where protein content is the amount of proteins in 400 µl of cell lysate determined by BCA.

Intracellular HO-1 staining

Cells were stained using the Perm/Wash buffer (BD Biosciences) according to the vendor's protocol and stained (4 °C, 40 minutes) with HO-1 primary polyclonal antibody (ADI-SPA 894, 1:200; Enzo Life Sciences) followed by secondary staining with goat anti-rabbit AlexaFluor 488 (1:400). Samples were analyzed using the Fortessa BD flow cytometer and data were proceeded in FACS Diva™ Software.

References

[1] Foresti, R.; Clark, J. E.; Green, C. J.; Motterlini, R. Thiol Compounds Interact with Nitric Oxide in Regulating Heme Oxygenase-1 Induction in Endothelial Cells. *J. Biol. Chem.* **1997**, *272* (29), 18411–18417.

[2] Mucha, O.; Podkalicka, P.; Czarnek, M.; Biela, A.; Mieczkowski, M.; Kachamakova-Trojanowska, N.; Stepniewski, J.; Jozkowicz, A.; Dulak, J.; Loboda, A. Pharmacological versus Genetic Inhibition of Heme Oxygenase-1 - The Comparison of Metalloporphyrins, ShRNA and CRISPR/Cas9 System. *Acta Biochim. Pol.* **2018**, *65* (2), 277–286. https://doi.org/10.18388/abp.2017_2542.
